# Atherosclerotic plaque locations may be related to different ischemic lesion patterns

**DOI:** 10.1186/s12883-020-01868-0

**Published:** 2020-07-30

**Authors:** Ho Geol Woo, Sung Hyuk Heo, Eui Jong Kim, Dae-il Chang, Tae Jin Song, Bum Joon Kim

**Affiliations:** 1 Departments of Neurology, Kyung Hee University College of Medicine, Seoul, South Korea; 2 Departments of Radiology, Kyung Hee University College of Medicine, Seoul, South Korea; 3 Department of Neurology, Seoul Hospital Ewha Womans University College of Medicine, Seoul, South Korea; 4 Department of Neurology, Asan Medical Center, University of Ulsan, Song-Pa, PO Box 145, Seoul, 138-600 South Korea

**Keywords:** Atherosclerosis, Ischemic stroke, Hemodynamics

## Abstract

**Background:**

Atherosclerosis of the internal carotid artery (ICA) is an important cause of ischemic stroke. Artery-to-artery embolism is the major stroke mechanism in patients with atherosclerotic carotid disease. This study hypothesized that the atherosclerotic ICA geometry and plaque location would be associated with lesion pattern in patients with acute ischemic stroke.

**Methods:**

Ischemic stroke patients with symptomatic proximal ICA disease (> 50% diameter stenosis) were enrolled. The carotid plaque location was divided into high-apical and low-body types. The geometric parameters of the ICA (angles between arteries) were measured, and ischemic lesion patterns were classified according to the number, location, and size of the lesions. Factors associated with plaque location and lesion pattern, dichotomized by size, were investigated.

**Results:**

Of the 93 acute ischemic stroke patients enrolled, 31 had high-apical and 62 had low-body plaques. Hyperlipidemia was more prevalent and the common carotid artery (CCA)-ICA angle was wider (167.7 ± 10.4° vs 162.3 ± 9.8°, *p* = 0.019) in patients with low-body than high-apical plaques. Low-body plaques were more frequently associated with small scattered or cortical lesions (54.8% vs. 32.3%, *p* = 0.040), whereas high-apical plaques were more frequently associated with large lesions having additional lesions (38.7% vs. 11.3%, *p* = 0.002). The presence of low-body plaques (odds ratio: 3.106, 95% confidence interval: 1.105–8.728, *p* = 0.032) was independently associated with the small lesion-only pattern.

**Conclusions:**

Low-body plaques are more frequently associated with small scattered lesions, whereas high-apical plaques are more frequently associated with large lesions having additional lesions. A wide CCA-ICA angle is associated with low-body plaque of the carotid artery.

## Background

Atherosclerosis of the carotid artery is one of the major causes of ischemic stroke, with artery-to-artery embolism being the major stroke mechanism in patients with atherosclerosis of the carotid artery [1]. Thromboembolism due to rupture of ulcerative plaque as well as the degree of stenosis has been especially associated with ischemic stroke in these patients [2, 3]. These risk factors, however, have not been associated with the distribution and size of these ischemic lesions. Blood flow may be an important factor affecting lesion size and pattern because it can influence the activation of platelets and the coagulation cascade and dislodge thrombi [4, 5].

Although atherosclerotic plaque of the carotid artery is associated with old age, male sex, and hyperlipidemia [1], these risk factors have not been associated with specific plaque locations in the carotid artery among individual patients. In addition to these general risk factors, hemodynamics may also affect the location of plaque within the carotid artery. For example, high wall shear stress (WSS) is associated with plaque formation at the apex of the carotid artery and low WSS with plaque formation at the bulb of the carotid artery [6, 7]. Moreover, the geometric properties of the carotid artery (i.e., bifurcation angle and radius), which are thought to regulate WSS, have been associated with plaque location in the carotid artery [8, 9]. Specifically, the nature and sizes of emboli from atherosclerotic stenotic lesions may depend on plaque location affecting the WSS inside the carotid artery. The present study therefore investigated the association of lesion patterns of acute ischemic stroke, as shown by diffusion-weighted imaging (DWI), with the location of carotid plaques.

## Methods

### Subjects

This study was a retrospective analysis of a prospectively collected database of acute ischemic stroke patients (within 7 days from stroke onset) between January 2013 and December 2017. Patients classified by the Trial of Org 10,172 in Acute Stroke Treatment (TOAST) as having large artery atherosclerosis with symptomatic proximal internal carotid artery (pICA) disease were enrolled [10]. Significant stenosis of the pICA was defined as > 50% diameter reduction to near-occlusion (artery beyond the stenosis has collapsed, but has remaining patency). Symptomatic pICA disease was considered present when significant stenosis of the pICA was the likely cause of index middle cerebral artery (MCA) or anterior cerebral artery (ACA) infarction [11, 12].

Patients were excluded if they had (1) embolic sources from the heart (e.g., atrial fibrillation or valvular heart disease) or the aorta, (2) other uncommon etiologies such as dissection or moyamoya disease, (3) tandem stenotic lesions at the intracranial ICA and MCA or ACA, or (4) results on contrast enhanced magnetic resonance angiography (MRA) that did not allow assessment of geometric properties (e.g., complete occlusion or poor MRA quality). The study protocol was approved by the institutional review board of Kyung Hee University Hospital, which waived the need for informed consent because of its retrospective nature.

### Clinical data and neuroimaging

Demographic characteristics, vascular risk factors, concurrent medications, and baseline laboratory results were obtained from the prospectively acquired stroke database. Hyperlipidemia was defined by patient having past history of hyperlipidemia, using a lipid-lowering agent including statins or patient diagnosed hyperlipidemia at admission for LDL-cholesterol levels [13].

On the day of admission, all patients underwent magnetic resonance imaging and MRA in the following sequence: DWI, fluid attenuated inversion recovery imaging, gradient echo imaging, T1- and T2-weighted imaging, and intracranial and extracranial contrast enhanced MRA.

The patterns of infarction on DWI were classified according to the number, location, and maximum diameter of lesions [14, 15]. Lesions were classified as single or multiple, and as large (≥15 mm) or small (< 15 mm) based on their maximum diameter. Single lesions were further classified as cortical (small), subcortical (small or large), or cortico-subcortical (large). Multiple lesions were classified as small scattered lesions or a large lesion with additional lesions. The topography of ischemic lesions according to vascular territory was determined with reference to published templates [16]. For further analysis, lesion pattern was dichotomized according to the presence or absence of a large lesion. Lesion location was divided in to cortical, subcortical and cortico-subcortical lesions.

### Carotid plaque and geometry

The presence and location of plaques and the degree of stenosis in the pICA were assessed on contrast enhanced MRA. Atherosclerotic plaques of the carotid artery were classified as being high-apical or low-body plaques. High-apical plaques were defined as plaques in the transitional zone of the bulb and the proximal cervical ICA segment, with or without the involvement of the body segment. Low-body plaques were defined as those in the transitional zone of the common carotid artery (CCA) and the bulb, and are located mainly in the lower body segment, with or without the involvement of the apical segment (Supplementary Figure S1). To differentiate among type of enlarged plaques, the location of the main plaque component and the level of the most severe stenosis were considered [7]. Two independent researchers evaluated the type of plaque and dichotomized the location to high-apical or low-body type. If any discrepancy exists, a consensus meeting was held to finalize the type of atherosclerosis, with more than three researchers and considering all the imaging modalities available.

The geometry of the carotid artery was quantitatively analyzed by a modification of previous methods [17]. The ICA-external carotid artery (ECA) angle was defined as the angle between the projections of the ICA0-ICA5 and ECA0-ECA5 vectors onto the bifurcation plane (Supplementary Figure S1). The CCA-ICA and CCA-ECA angles were defined similarly. Moreover, ICA planarity was defined as the angle between the out-of-plane components of the CCA and ICA vectors. The ICA-to-CCA diameter ratio was calculated as the ICA5 diameter divided by the CCA5 diameter.

Geometric factors that could affect the risk of embolism or vulnerability of plaque were also evaluated. These factors included 1) the degree of stenosis measured by North American Symptomatic Carotid Endarterectomy Trial (NASCET) criteria; 2) the presence of ulcer, defined as a niche in the plaque surface > 2 mm in depth on contrast enhanced extracranial MRA [18]; 3) carotid webs, defined as thin intraluminal filling defects along the posterior wall of the carotid bulb in oblique sagittal reformatted image on contrast enhanced extracranial MRA [19]; and 4) ICA kinking, defined as an extreme form of tortuous ICA with angulation of the vessel’s axis ≤ 90° [20].

### Statistical methods

Demographic characteristics, vascular risk factors, concurrent medications, laboratory findings, geometrical factors, and ischemic lesion patterns were compared in patients grouped by plaque location. Pearson chi-square tests, independent *t-*tests, and Mann-Whitney U-tests were used as appropriate. In addition, clinical and imaging characteristics were compared according to different lesion patterns and location on DWI. Multivariable binary logistic regression analysis was performed to investigate the independent association between lesion patterns (small lesions only vs. large lesion) and the type of plaque and the geometric properties of the carotid bifurcation. Odds ratios (ORs) were calculated, along with 95% confidence intervals (95% CIs). A *p* value < 0.05 was considered statistically significant. All statistical analyses were performed using SPSS 22.0 for Windows (IBM Corp. Armonk, NY, USA).

## Results

Of the 3061 patients with acute ischemic stroke between January 2013 and December 2017, 657 (21.5%) were classified as having large artery atherosclerosis according to the TOAST classification. Among the latter, 117 (17.8%) patients had symptomatic pICA disease. After excluding patients with complete occlusion of the carotid artery, those with no DWI or MRA data, and those with poor MRA quality, the final study population included 93 patients.

The 93 patients included 75 (80.6%) men and 18 (19.4%) women of mean age 72.0 ± 8.3 years. Considering the location of the plaque, 43 (46.2%) patients showed atherosclerosis extending from the proximal to the distal area, but in most of the cases atherosclerosis was dominant in one area. Only 8 patients showed discrepancy between the two researchers. Finally, after a consensus meeting, 31 (33.3%) had high-apical and 62 (66.7%) had low-body plaque.

### Characteristics of patients with high-apical and low-body plaques

Table 1 shows that history of hyperlipidemia was significantly more frequent in patients with low-body than high-apical plaques (*p* = 0.022). The CCA-ICA angle was significantly wider in patients with low-body than high-apical plaques (167.7 ± 10.4° vs. 162.3 ± 9.8°, *p* = 0.019; Table 2). Multivariable analysis showed that, relative to high-apical plaques, wider CCA-ICA angle (OR: 1.061, 95% CI: 1.010–1.115, *p* = 0.018) and narrower ICA-ECA angle (OR: 0.961, 95% CI: 0.925–0.999, *p* = 0.043) were independently associated with low-body plaques (Supplementary Table S1).

**Table 1:** Baseline demographic and clinical characteristics of patients with high-apical and low-body plaques

Variables	High-apical type (*n* = 31)	Low-body type (*n* = 62)	*P* value
Age, y	72.7 ± 6.8	71.7 ± 9.0	0.588
Male sex	23 (74.2)	52 (83.9)	0.265
Hypertension	25 (80.6)	48 (77.4)	0.721
Diabetes mellitus	12 (38.7)	21 (33.9)	0.646
Hyperlipidemia	15 (48.4)	45 (72.6)	0.022
Smoking	17 (54.8)	36 (58.1)	0.767
History of stroke or TIA	11 (35.5)	15 (24.2)	0.253
Previous medication			
Antiplatelet	12 (38.7)	30 (48.4)	0.377
Statin	9 (29.0)	25 (40.3)	0.287
Laboratory findings			
WBC, per mm^2^	9144.2 ± 5383.2	8514.7 ± 2669.0	0.452
Hematocrit	38.8 ± 6.2	40.6 ± 4.9	0.119
Platelet, × 10^3^/mm^2^	264.0 ± 115.2	236.8 ± 65.8	0.152
Glucose, mg/dL	124.9 ± 36.8	137.3 ± 53.6	0.262
Glycated hemoglobin (HbA1c)	6.3 ± 1.1	6.5 ± 1.4	0.610
Total cholesterol, mg/dL	159.5 ± 55.9	170.7 ± 51.4	0.341
HDL cholesterol, mg/dL	37.7 ± 8.8	40.7 ± 11.4	0.202
Triglyceride, mg/dL	132.6 ± 80.1	146.3 ± 104.4	0.523
LDL cholesterol, mg/dL	106.4 ± 41.7	108.8 ± 39.9	0.785
BUN, mg/dL	17.0 ± 6.0	18.6 ± 7.4	0.303
Creatinine, mg/dL	0.9 ± 0.3	1.0 ± 0.7	0.679
CRP, mg/dL	1.2 ± 3.9	0.7 ± 2.1	0.393

Values are expressed as number (%), mean ± standard deviation, or median [interquartile range].

Abbreviations: *BUN* blood urea nitrogen, *CRP* C-reactive protein, *HDL* high-density lipoprotein, *LDL* low-density lipoprotein, *TIA* transient ischemic attack, *WBC* white blood cell

**Table 2:** Carotid geometry and lesion patterns among patients with high-apical and low-body plaques

	High-apical type (*n* = 31)	Low-body type (*n* = 62)	*P* value
Carotid geometry			
ICA-ECA angle, °	28.4 ± 14.9	22.8 ± 10.0	0.067
CCA-ICA angle, °	162.3 ± 9.8	167.7 ± 10.4	0.019
CCA-ECA angle, °	168.8 ± 12.4	169.1 ± 12.1	0.869
ICA-to-CCA diameter ratio	0.56 ± 0.13	0.55 ± 0.12	0.832
ICA planarity	17.0 ± 8.9	18.1 ± 10.7	0.625
ICA stenosis severity (NASCET)	69.2 ± 11.4	64.6 ± 13.6	0.105
Kinking of ICA	2 (6.5)	3 (4.8)	0.999
Ulceration of plaque	16 (51.6)	35 (56.5)	0.658
Carotid web	0	0	N/A
Lesion pattern on DWI			
Small (< 15 mm) single cortical lesion	2 (6.5)	2 (3.2)	0.598
Small (< 15 mm) single subcortical lesion	1 (3.2)	4 (6.5)	0.662
Small (< 15 mm) multiple scattered lesions	10 (32.3)	34 (54.8)	0.040
Large single cortico-subcortical lesion	5 (16.1)	9 (14.5)	0.999
Large single subcortical lesion	1 (3.2)	6 (9.7)	0.418
Large lesion with additional lesions	12 (38.7)	7 (11.3)	0.002

Values are expressed as number (%) or mean ± standard deviation.

Abbreviations: *CCA* common carotid artery, *DWI* diffusion-weighted imaging, *ECA* external carotid artery, *ICA* internal carotid artery, *N/A* not available, *NASCET* North American Symptomatic Carotid Endarterectomy Trial

### Carotid geometry and lesion pattern

Small scattered lesions were more frequently associated with low-body plaques (54.8% vs. 32.3%, *p* = 0.040), whereas large lesions accompanied by additional lesions were more frequently associated with high-apical plaques (38.7% vs. 11.3%, *p* = 0.002; Table 2). An analysis of clinical and imaging factors according to lesion pattern and location on DWI found that there was no difference except the prevalence of low-body plaque (Supplementary Table S2 and Supplementary Table S3, respectively). Multivariable analysis comparing the small lesions only pattern found that low-body plaque was the only factor independently associated with the small lesions only pattern (OR: 3.106, 95% CI: 1.105–8.728, *p* = 0.032; Table 3).

**Table 3:** Multivariable analysis of factors associated with the small lesion-only type

	Model 1, unadjusted Odds ratio (95% CI)	Model 2, ^a^Odds ratio (95% CI)	Model 3, ^b^Odds ratio (95% CI)
ICA-ECA angle, ° CCA-ICA angle, ° CCA-ECA angle, ° Low-body plaque	0.998 (0.965–1.032) 0.978 (0.940–1.018) 1.021 (0.986–1.057) 2.571 (1.041–6.087)*	0.999 (0.959–1.040) 0.970 (0.927–1.015) 1.029 (0.987–1.073) 3.058 (1.107–8.449)*	0.999 (0.960–1.042) 0.970 (0.927–1.016) 1.027 (0.984–1.072) 3.106 (1.105–8.728)*

*p < 0.05

^a^Adjusted for hypertension, low-density lipoprotein cholesterol, and white blood cell count.

^b^Adjusted for age, male sex, hypertension, low-density lipoprotein cholesterol, and white blood cell count.

Abbreviations: *CCA* common carotid artery, *CI* confidence interval, *ECA* external carotid artery, *ICA* internal carotid artery

## Discussion

The present study found that the CCA-ICA angle was wider in patients with low-body than high-apical plaques. These low-body plaques showed a greater association with small scattered lesions and were independently associated with having only small lesions of diameter < 15 mm. Patients with high-apical plaques had a narrower CCA-ICA angle and were more likely to have large ischemic lesions.

In agreement with previous findings [7], the present study found that a history of hyperlipidemia was associated with low-body plaques. Hyperlipidemia, however, acts systemically, making its association with the specific location of atherosclerosis unclear. Local factors such as vascular geometry may influence the site of atherosclerosis development. A study of the computational fluid dynamics of the carotid artery found that the bifurcation angle was significantly negatively correlated with WSS in the inner and outer walls of the ICA, which may have been due to a loss of energy [21]. A decrease in the CCA-ICA angle may be associated with a more rapid decrease in WSS in the inner than in the outer wall of the ICA [21]. These may explain our result showing that a decreased CCA-ICA angle was independently associated with the presence of high-apical plaques. Of our 31 patients with high-apical plaques, 25 had plaques located in the inner curvature and a more rapid decrease in WSS. Furthermore, a second angle is present in patients with a decreased CCA-ICA angle, leading to low WSS in the inner curvature that is prone to atherosclerosis development [22].

**Fig. 1:**
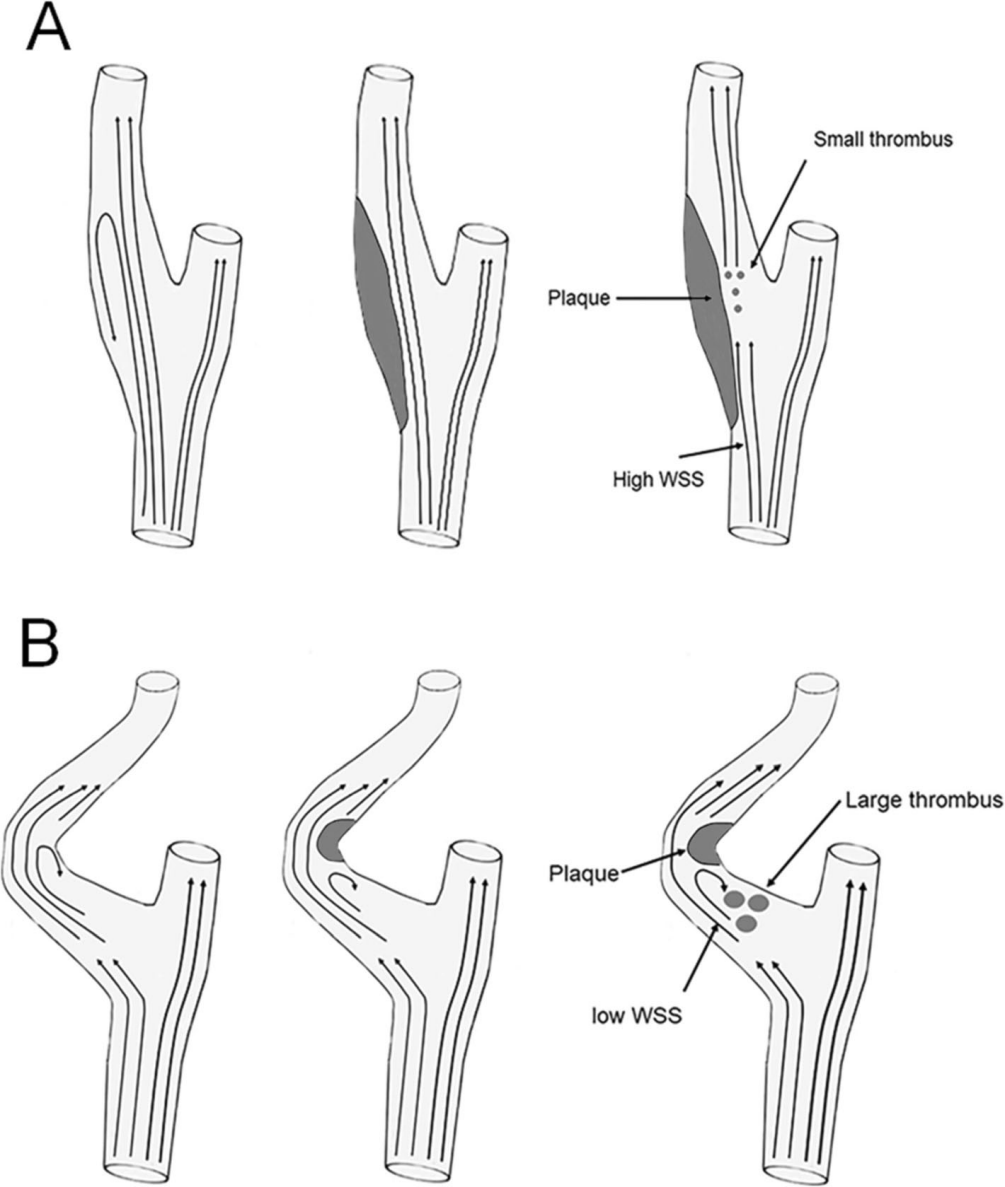
Schematic drawing of plaque and thrombus formation in the carotid artery

Our results also showed that the lesion patterns on DWI in acute ischemic stroke patients with symptomatic pICA disease were associated with plaque location, but not with the geometry of the ICA or the presence of ulcer. Small scattered lesions were associated with low-body plaques of the ICA, whereas the presence of a large lesion with additional lesions was associated with high-apical plaques. Dichotomization of lesion patterns according to the presence or absence of a large lesion resulted in low-body plaques being independently associated with the small lesion-only pattern. In high-apical plaques, the bulb is proximal to the stenotic portion with a back flow causing fluid stagnation and thrombus formation, such as in the appendage of the left atrium [23, 24]. This may cause a large thrombus inside the bulb area, resulting in a large-sized infarction [25]. By contrast, low-body plaques increase the flow velocity at the bulb area. The high speed flow may induce shear dependent platelet activation and generate multiple small thrombi, resulting in small scattered lesions (Fig. 1) [26].

The present study had several limitations. First, its retrospective nature may have caused a selection bias. Second, the number of patients was small, under-powering some of our results, especially the lesion patterns. Third, geometry was analyzed based on two-dimensional anteroposterior images of three-dimensionally reconstructed MRA images. Though three-dimensional analysis of the vascular geometry might strengthen our results, two-dimensional analysis is more practical. Fourth, the present study did not analyze the state of intracerebral hemodynamics, the activation of the collateral circulation, or the presence of microembolic signals. Finally, this study did not pathologically assess the composition and vulnerability of the plaques or the size and composition of the embolus resulting from each stroke mechanism. Despite these limitations, our results suggest the importance of vascular geometry in the development of acute stroke in patients with symptomatic pICA disease.

## Conclusion

The CCA-ICA angle may be a factor determining the location of atherosclerotic plaques of the carotid artery, probably altering hemodynamics. Differences in the locations of carotid plaques may explain, at least in part, inter-individual differences in the location of lesions in acute ischemic stroke in patients with symptomatic pICA disease.

## Supplementary information

**Additional file 1.**

**Additional file 2: Table S1.** Multivariable analysis of factors associated with low-body plaques. **Table S2.** Association independent factors with lesion pattern on diffusion-weighted imaging. **Table S3.** Association independent factors with lesion location including cortical, subcortical, and cortico-subcortical lesions.

## Data Availability

Our dataset was prospectively collected registry for stroke patients in our hospital. The datasets used and/or analyzed during the current study are available from the corresponding author on reasonable request. Public access to the datasets is closed and administrative permission was needed to access and use our datasets.

## Abbreviations

WSS: Wall shear stress
DWI: Diffusion-weighted imaging
TOAST: Trial of Org 10,172 in Acute Stroke Treatment
pICA: proximal internal carotid artery
MCA: Middle cerebral artery
ACA: Anterior cerebral artery
MRA: Magnetic resonance angiography
CCA: Common carotid artery
ECA: External carotid artery
NASCET: North American Symptomatic Carotid Endarterectomy Trial
ORs: Odds ratios
CIs: Confidence intervals
