# Supplementary figures and images for: Atherosclerotic plaque locations may be related to different ischemic lesion patterns
Source: BMC Neurol. 2020 Jul 30;20:288. doi: 10.1186/s12883-020-01868-0 (PMC7391573; doi:10.1186/s12883-020-01868-0)

A

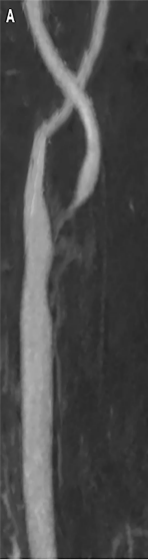

B

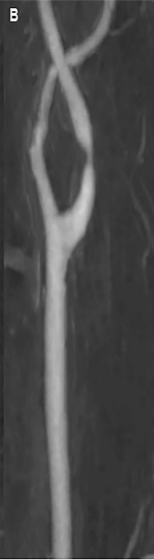

C

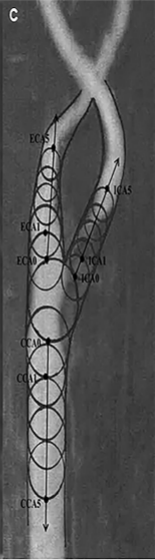

D

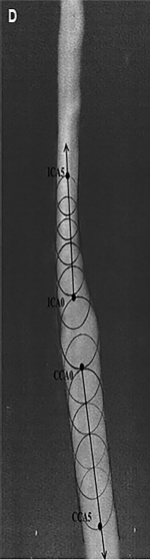

Supplement: Supplementary file 1 — Additional file 1. [file 12883_2020_1868_MOESM1_ESM.pdf]
