# Supplementary material for: Atherosclerotic plaque locations may be related to different ischemic lesion patterns
Source: BMC Neurol. 2020 Jul 30;20:288. doi: 10.1186/s12883-020-01868-0 (PMC7391573; doi:10.1186/s12883-020-01868-0)
Supplement: Supplementary file 2 — Additional file 2: Table S1. Multivariable analysis of factors associated with low-body plaques. Table S2. Association independent factors with lesion pattern on diffusion-weighted imaging. Table S3. Association independent factors with lesion location including cortical, subcortical, and cortico-subcortical lesions. [file 12883_2020_1868_MOESM2_ESM.docx]

**Additional file**

**Supplementary Table 1.** Multivariable analysis of factors associated with low-body plaques.

|  | Model 1, Unadjusted,  Odd ratio (95% CI) | Model 2,  §Odd ratio (95% CI) | Model 3,  †Odd ratio (95% CI) |
| --- | --- | --- | --- |
| ICA-ECA angle**, °**  CCA-ICA angle**, °**  CCA-ECA angle**, °** | 0.963 (0.929–0.999)*  1.055 (1.007–1.104)*  1.002 (0.967–1.039) | 0.959 (0.924–0.996)*  1.061 (1.010–1.114)*  1.005 (0.968–1.042) | 0.961 (0.925–0.999)*  1.061 (1.010–1.115)*  1.002 (0.964–1.041) |

* *p* < 0.05.

§ Adjusted for hyperlipidemia.

† Adjusted for male sex, age, and hyperlipidemia.

Abbreviations: CCA, common carotid artery; CI, confidence interval; ECA, external carotid artery; ICA, internal carotid artery;

**Supplementary Table 2.** Association independent factors with lesion pattern on diffusion-weighted imaging.

|  | Lesion pattern on diffusion-weighted imaging | | | | | | *p* value |
| --- | --- | --- | --- | --- | --- | --- | --- |
|  | Small single cortical lesion (n =4) | Small multiple scattered lesion (n =44) | Small single subcortical lesion (n =5) | Large single subcortical lesion (n =7) | Large single cortico-subcortical lesion (n =14) | Large lesion with additional lesions (n =19) |  |
| Age, y | 73.2 ± 6.1 | 73.5 ± 7.7 | 65.0 ± 5.0 | 72.8 ± 11.1 | 68.2 ± 8.4 | 72.5 ± 8.7 | 0.147 |
| Male sex | 10 (25.0) | 39 (88.6) | 5 (100.0) | 4 (57.1) | 11 (78.6) | 15 (78.9) | 0.971 |
| Hypertension | 4 (100.0) | 33 (75.0) | 1 (20.0) | 7 (100.0) | 11 (78.6) | 17 (89.5) | 0.464 |
| Diabetes mellitus | 1 (25.0) | 16 (36.4) | 3 (60.0) | 2 (28.6) | 3 (21.4) | 8 (42.1) | 0.885 |
| Hyperlipidemia | 3 (75.0) | 31 (70.5) | 2 (40.0) | 4 (57.1) | 9 (64.3) | 11 (57.9) | 0.270 |
| Smoking | 1 (25.0) | 26 (59.1) | 5 (100.0) | 4 (57.1) | 7 (50.0) | 10 (52.6) | 0.974 |
| History of stroke or TIA | 1 (25.0) | 14 (31.8) | 3 (60.0) | 0 | 4 (28.6) | 4 (21.1) | 0.417 |
| Geometric parameters |  |  |  |  |  |  |  |
| ICA-ECA angle, ° | 31.0 ± 14.8 | 23.5 ± 11.0 | 29.2 ± 7.8 | 21.5 ± 9.3 | 29.0 ± 17.1 | 23.1 ± 11.4 | 0.461 |
| CCA-ICA angle, ° | 162.2 ± 2.5 | 164.9 ± 10.0 | 166.8 ± 10.1 | 168.1 ± 10.2 | 167.7 ± 14.0 | 166.6 ± 10.5 | 0.894 |
| CCA-ECA angle, ° | 166.7 ± 16.1 | 171.4 ± 10.7 | 164.0 ± 4.7 | 169.4 ± 6.5 | 163.2 ± 18.5 | 169.6 ± 11.1 | 0.328 |
| ICA-to-CCA diameter ratio | 0.58 ± 0.15 | 0.55 ± 0.13 | 0.57 ± 0.06 | 0.59 ± 0.09 | 0.55 ± 0.12 | 0.55 ± 0.13 | 0.982 |
| ICA planarity | 21.5 ± 4.3 | 16.8 ± 9.7 | 15.8 ± 8.0 | 20.7 ± 9.7 | 21.0 ± 14.2 | 16.2 ± 9.1 | 0.629 |
| ICA stenosis severity (NASCET) | 64.1 ± 9.9 | 65.1 ± 14.0 | 64.5 ± 13.1 | 71.1 ± 10.0 | 60.9 ± 13.0 | 71.3 ± 11.1 | 0.246 |
| Kinking of ICA | 0 | 2 (4.5) | 0 | 1 (14.3) | 1 (7.1) | 1 (5.3) | 0.633 |
| Ulceration of plaque | 1 (25.0) | 28 (63.6) | 1 (20.0) | 5 (71.4) | 6 (42.9) | 10 (52.6) | 0.559 |
| Low-body plaque | 2 (50.0) | 34 (77.3) | 4 (80.0) | 6 (85.7) | 9 (64.3) | 7 (36.8) | 0.020 |

Data are expressed as the mean ± SD, or n (%).

P-value by ANOVA and chi-square test.

Abbreviations: CCA, common carotid artery; ECA, external carotid artery; ICA, internal carotid artery; NASCET, North American Symptomatic Carotid Endarterectomy Trial; TIA, transient ischemic attack;

**Supplementary Table 3.** Association independent factors with lesion location including cortical, subcortical, and cortico-subcortical lesions.

|  | Lesion location | | | *p* value |
| --- | --- | --- | --- | --- |
|  | Cortical lesion  (n =48) | Subcortical lesion  (n =12) | Cortico-subcortical lesion (n =33) |  |
| Age, y | 73.5 ± 7.5 | 69.5 ± 9.6 | 70.7 ± 8.7 | 0.185 |
| Male sex | 40 (83.3) | 9 (75.0) | 26 (78.8) | 0.575 |
| Hypertension | 37 (77.1) | 8 (66.7) | 28 (84.8) | 0.488 |
| Diabetes mellitus | 17 (35.4) | 5 (41.7) | 11 (33.3) | 0.895 |
| Hyperlipidemia | 34 (70.8) | 6 (50.0) | 20 (60.6) | 0.289 |
| Smoking | 27 (56.3) | 9 (75.0) | 17 (51.5) | 0.798 |
| History of stroke or TIA | 15 (31.3) | 3 (25.0) | 8 (24.2) | 0.480 |
| Geometric parameters |  |  |  |  |
| ICA-ECA angle, ° | 24.1 ± 11.3 | 24.7 ± 9.2 | 25.6 ± 14.2 | 0.866 |
| CCA-ICA angle, ° | 164.7 ± 9.6 | 167.5 ± 9.7 | 167.1 ± 11.9 | 0.515 |
| CCA-ECA angle, ° | 171.0 ± 11.1 | 167.1 ± 6.2 | 166.9 ± 14.8 | 0.286 |
| ICA-to-CCA diameter ratio | 0.55 ± 0.13 | 0.58 ± 0.08 | 0.55 ± 0.13 | 0.786 |
| ICA planarity | 17.2 ± 9.4 | 18.6 ± 9.0 | 18.2 ± 11.6 | 0.871 |
| ICA stenosis severity (NASCET) | 65.1 ± 13.6 | 68.3 ± 11.3 | 66.9 ± 12.9 | 0.684 |
| Kinking of ICA | 2 (4.2) | 1 (8.3) | 2 (6.1) | 0.674 |
| Ulceration of plaque | 29 (60.4) | 6 (50.0) | 16 (48.5) | 0.278 |
| Low-body plaque | 36 (75.0) | 10 (83.3) | 16 (48.5) | 0.021 |

Data are expressed as the mean ± SD, or n (%).

P-value by ANOVA and chi-square test.

Abbreviations: CCA, common carotid artery; ECA, external carotid artery; ICA, internal carotid artery; NASCET, North American Symptomatic Carotid Endarterectomy Trial; TIA, transient ischemic attack;
